# A Vaccinia Virus Recombinant Transcribing an Alphavirus Replicon and Expressing Alphavirus Structural Proteins Leads to Packaging of Alphavirus Infectious Single Cycle Particles

**DOI:** 10.1371/journal.pone.0075574

**Published:** 2013-10-09

**Authors:** Juana M. Sánchez-Puig, María M. Lorenzo, Rafael Blasco

**Affiliations:** Departamento de Biotecnología, Instituto Nacional de Investigación y Tecnología Agraria y Alimentaria (I.N.I.A.), Madrid, Spain; Kantonal Hospital St. Gallen, Switzerland

## Abstract

Poxviruses and Alphaviruses constitute two promising viral vectors that have been used extensively as expression systems, or as vehicles for vaccine purposes. Poxviruses, like vaccinia virus (VV) are well-established vaccine vectors having large insertion capacity, excellent stability, and ease of administration. In turn, replicons derived from Alphaviruses like Semliki Forest virus (SFV) are potent protein expression and immunization vectors but stocks are difficult to produce and maintain. In an attempt to demonstrate the use of a Poxvirus as a means for the delivery of small vaccine vectors, we have constructed and characterized VV/SFV hybrid vectors. A SFV replicon cDNA was inserted in the VV genome and placed under the control of a VV early promoter. The replicon, transcribed from the VV genome as an early transcript, was functional, and thus capable of initiating its own replication and transcription. Further, we constructed a VV recombinant additionally expressing the SFV structural proteins under the control of a vaccinia synthetic early/late promoter. Infection with this recombinant produced concurrent transcription of the replicon and expression of SFV structural proteins, and led to the generation of replicon-containing SFV particles that were released to the medium and were able to infect additional cells. This combined VV/SFV system in a single virus allows the use of VV as a SFV delivery vehicle *in vivo*. The combination of two vectors, and the possibility of generating *in vivo* single-cycle, replicon containing alphavirus particles, may open new strategies in vaccine development or in the design of oncolytic viruses.

## Introduction

Virus-based expression systems have been derived from members of diverse virus families, including widely different RNA and DNA viruses. Among those, Vaccinia virus (VV), the representative member of the Poxviridae, constitutes an extensively used protein expression and vaccine vector. In addition to many beneficial characteristics for vaccine use, a major advantage of VV vectors is their large DNA genome that provides considerable insertion capacity, thus allowing the expression of large and/or multiple genes. In contrast, Alphavirus-based vectors are expression systems which are smaller in size and insertion capacity, but constitute attractive vaccine candidates shown to induce strong immune responses. For reviews on Alphavirus vectors see [1], [2], [3], [4].

Alphaviruses are members of the family *Togaviridae,* whose genome is a positive-sense single-stranded RNA molecule of approximately 12 kb. After infection, the 5′ two-thirds of the incoming genome is translated, producing the viral replicase nonstructural proteins (nsP1–4). Next, the replicase synthesizes negative-sense copies of the genome, which serve as templates for both progeny genomes and for transcription of an mRNA from the internal subgenomic promoter [5].

Self-amplifying Alphavirus replicons are derived from the viral genome by replacing the genomic region coding for the viral structural proteins by a foreign gene [6]. Therefore, such replicons consist of a single RNA molecule which, when transfected into cells, is translated into the viral replicase, which amplifies the replicon and transcribes a subgenomic RNA encompassing the foreign gene. To facilitate introduction in cells, replicon RNA molecules have been packaged by Alphavirus structural proteins provided by a helper replicon *in trans*. By packaging the replicon RNA into SFV particles, single-cycle virus particles are produced, that can be subsequently used to introduce the replicon into new cells. Over the last years, considerable experience has been accumulated in the use of alphavirus-based systems for immunization.

Since the design of Alphavirus replicons, a number of strategies have been used to introduce the replicon into cells. Original systems relied on transfection of replicons transcribed *in vitro* using T7 or SP6 polymerases. Subsequently, other methods for intracellular delivery of Alphavirus replicons have been developed, including transcription from transfected plasmid DNA [7] [8], [9] or expression from baculovirus [10].

In this work we have sought to use vaccinia virus as a vehicle capable of delivering and packaging an alphavirus replicon within cells. Using this strategy, a Vaccinia virus/SFV combined vector can potentially be used as a single immunizing agent.

## Results

### Coinfection of Cells with Vaccinia and SFV Particles

**Figure 1:**
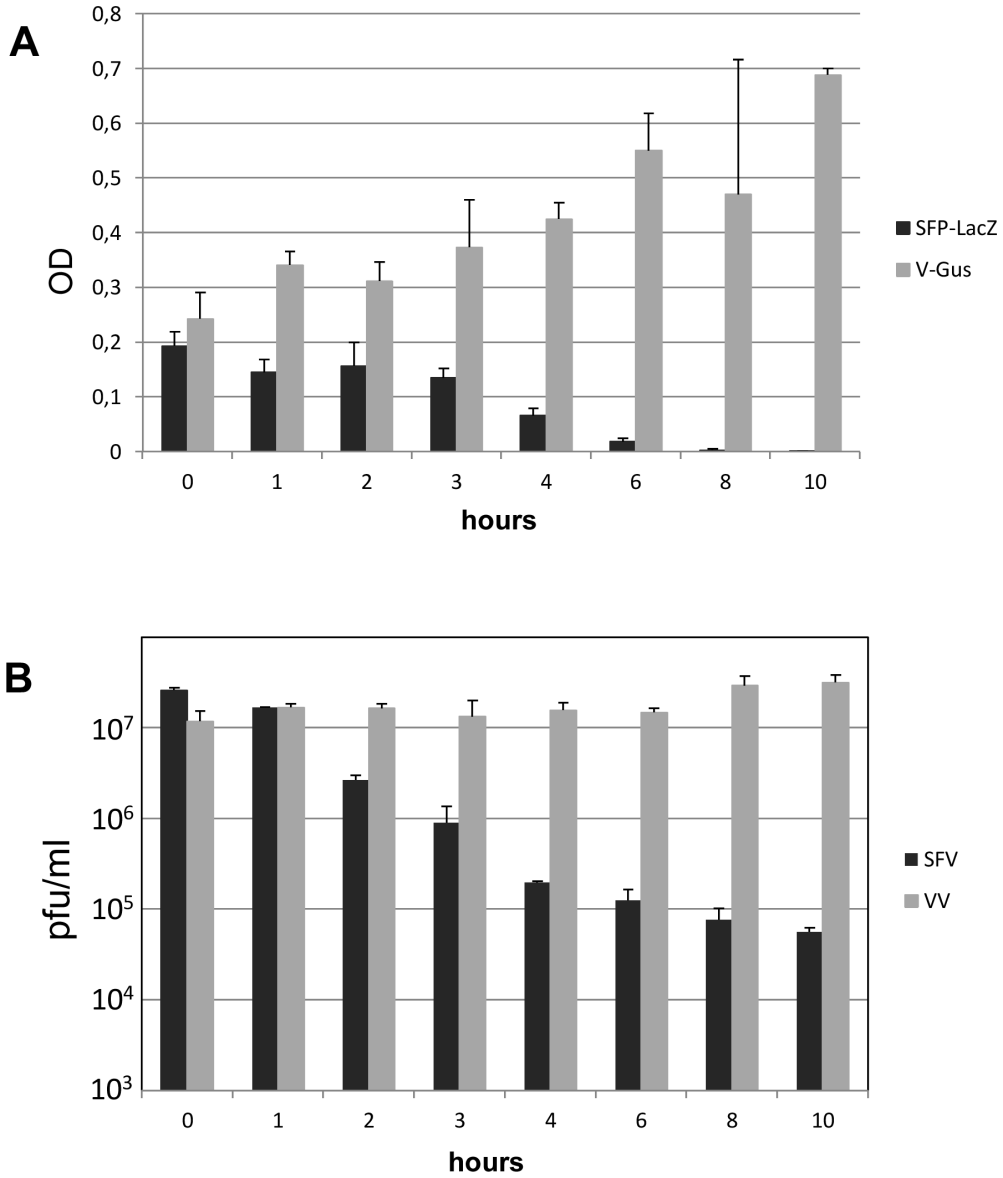
Coinfection of VV and SFV. **A)** BSC-1 cells were infected with VV expressing β–Glucuronidase and coinfected at different times with SFPs containing a SFV replicon with the β–Galactosidase gene. Both infections were carried out at a moi of 5 pfu/cell. 48 hours after the first infection, cells were lysed, and the β-galactosidase and β-glucuronidase activities in extracts corresponding to 10^3^ cells were assayed. **B)** BSC cells were infected with VV and then infected with SFV at different times post-vaccinia infection. At 48 hours, titers of Vaccinia virus were obtained by plaquing lysed cells (grey bars), and SFV titers were determined from culture media (black bars).

To ascertain whether VV and SFV replication cycles are compatible, i.e., can take place concurrently in the same cells, we carried out coinfections of cells with vaccinia virus and SFV replicons. In a first experiment, we used a vaccinia virus β–Glucuronidase recombinant and single-cycle SFV particles harboring the β–Galactosidase gene, and gene expression mediated by each system was measured. To compensate for kinetic differences between the two systems, SFV infections were started at different times after VV infection. After 48 hours, expression of β–Galactosidase and β–Glucuronidase were quantitated by measuring enzymatic activity with the specific substrates ONPG and PNG, respectively (Fig. 1A). Simultaneous infection with the two viruses resulted in significant amounts of β–Galactosidase and β–Glucuronidase, indicating that replication and gene expression of the two viruses were compatible. Notably, SFV-directed expression of β–Galactosidase was unaffected or enhanced in cells coinfected with vaccinia virus, provided that SFV infection was initiated within the first two hours after vaccinia infection. Also, virus production from coinfected cultures was within the normal ranges for both SFV and VV (Fig. 1B). Those results did not reveal any interference in the replication cycle of the two viruses, and indicate that the complete replication cycle of both viruses can proceed simultaneously.

### SFV Replicons can be Transcribed in Vaccinia Virus-infected Cells

**Figure 2:**
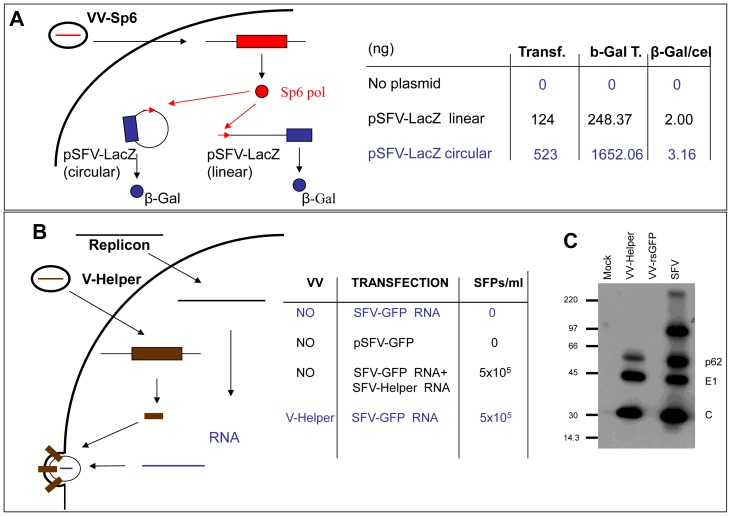
Transcription and packaging of the SFV replicon in VV-infected cells. **A)** BSC-1 cells were infected with vaccinia virus expressing SP6 RNA polymerase (VV-Sp6) and subsequently transfected with plasmid pSFV-LacZ. At 48 hours, cells were either stained for β-galactosidase by addition of X-Gal to the cultures or lysed to assay β-galactosidase activity. Transf.: Number of β-galactosidase positive cell in a 24-well plate well. β-Gal T: β-galactosidase in 10^5^ cells (pg). β-Gal/cel: ratio of β-galactosidase activity per cell. **B)** Packaging of replicon RNA by SFV structural proteins expressed from V-Helper. BHK-21 cells were transfected with plasmid pSFV-GFP, or with *in vitro* transcribed RNA from pSFV-GFP linearized with SpeI, and mock infected or infected with V-Helper at a moi of 5 pfu/cell. Column SFPs/ml shows the titers of SFPs in the culture media at 48 h post-infection. C) Western blot analysis of cell extracts infected with the viruses indicated at the top. The positions of SFV mature structural proteins p62, E1 and C are indicated.

In the SFV-based expression system originally described by Liljestrom and Garoff [6], [11] a capped and polyadenilated replicon RNA is generated from a linearized plasmid by *in vitro* transcription using SP6 RNA polymerase. However, generating a functional replicon from the VV genome would require transcription to take place in the cytoplasm of VV-infected cells. To examine if SFV replicons can be produced by SP6 RNA polymerase in VV-infected cells, plasmid pSFV-LacZ, in which the SFV replicon is placed downstream of the SP6 promoter, was transfected in cells previously infected with a vaccinia virus recombinant expressing SP6 RNA polymerase. 36 hours after transfection, β–Galactosidase activity was detected in the cell cultures (Fig. 2A). Interestingly, transfection of both linearized or closed circular plasmid resulted in β–Galactosidase expressing cells. In contrast, no β–Galactosidase was detected in parallel cultures transfected with pSFV-LacZ and infected with WR virus. The number of β–Galactosidase positive cells was higher after transfection of the uncut plasmid, probably revealing the higher efficiency of transfection, its amplification and/or the higher stability of the circular plasmid relative to the linearized plasmid inside cells. Those results indicate that functional SFV replicons can be generated in transfected cells by SP6 transcription. In addition, the fact that circular plasmids induced β–Galactosidase expression suggests that a functional 3′ end of the replicon RNA was generated inside cells after SP6 polymerase transcription.

### SFV Replicons can be Packaged by SFV Structural Proteins Provided *in trans* by Vaccinia Virus

A VV recombinant expressing the SFV structural proteins was constructed by inserting the complete set of SFV structural genes in the VV genome, downstream of the F13L gene and under the control of a synthetic vaccinia early/late promoter. To avoid any homology between the sequence inserted in vaccinia and the replicon, the bare coding sequence of the SFV structural proteins was used to insert in the VV genome. Therefore, the ensuing transcript would lack 5′ and 3′ non-coding sequences normally present in genomic (plus strand) SFV RNA or in SFV subgenomic RNA. The VV recombinant isolated was termed V-Helper and was shown to express SFV structural proteins by immunoblotting (Fig. 2C). To test the ability of the proteins expressed by V-Helper to package SFV replicon RNA, BHK-21 cells were transfected with an *in vitro* transcribed SFV-GFP replicon RNA, and then infected with V-Helper. The titer of single-cycle SFPs in cell supernatants after 36 hours was estimated by infecting fresh BHK cell monolayers. Titers were in the range of 10^5^ to 10^6^ SFPs/ml, and were similar to those obtained when co-transfecting *in vitro* transcribed replicon and SFV-Helper RNAs (Fig. 2B). An additional proof of the packaging ability of SFV structural proteins expressed by V-Helper was obtained by passaging single-cycle SFPs on monolayers of V-Helper- infected cells, which typically produced titers in the range of 10^6^ SFP/ml. Those results indicate that SFV replicons can be packaged into infective particles within vaccinia virus infected cells using packaging proteins provided *in trans* by a vaccinia-virus recombinant.

### SFV Replicons can be Generated as Early VV Transcripts

The compatibility of vaccinia virus and SFV replication opened the possibility to generate SFV replicons as VV early transcripts. With this objective, we inserted the complete SFV replicon cDNA into the VV thymidine kinase locus, placing the 5′ end of the replicon immediately downstream of the normal TK promoter. To facilitate complete transcription of the replicon, we mutagenized a vaccinia early transcription termination sequence (TTTTTnT) that was present in the non-structural region of the replicon sequence.

**Figure 3:**
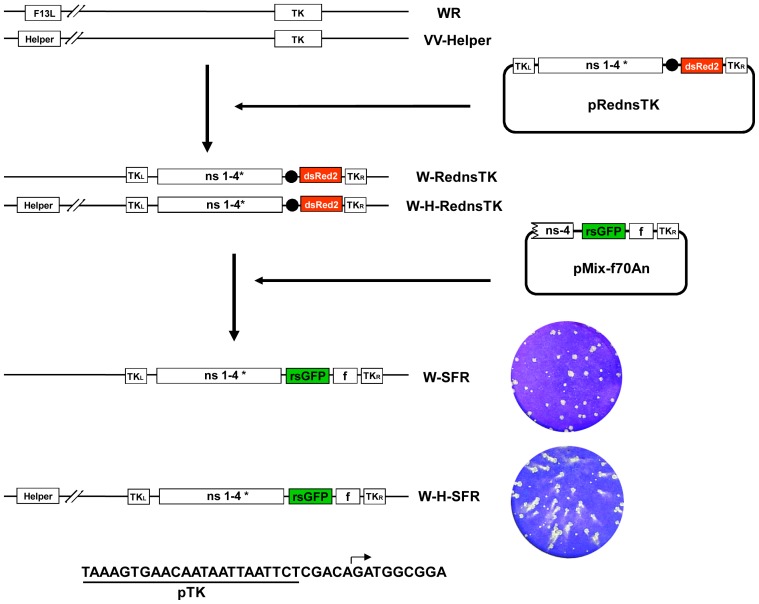
Construction of VV containing a SFV replicon. Upper panel: Schematic representation of the virus genome, indicating the F13L and the TK loci. Viruses W-RednsTK and W-H-RednsTK were obtained by recombination of VV WR and VV-Helper, respectively, with plasmid pRednsTK. Viruses W-SFR and W-H-SFR were obtained by recombination of W-RednsTK and W-H-RednsTK, respectively, with plasmid pMix-f70An. Boxes labeled TKL and TKR denote the left and right recombination flanks or the TK gene. Boxes ns1–4* correspond to the genomic region coding the non-structural SFV proteins with an early VV transcription termination signal mutated. Black circle: VV synthetic early/late promoter. dsRed: red fluorescent protein gene. Helper: SFV genes for structural proteins, inserted downstream of the F13L gene under the control of a VV synthetic early/late promoter. rsGFP: green fluorescent protein gene. f: sequence from the 3′end of the SFV replicon, including 70 nucleotides adjacent to the PolyA and a 70 nt PolyA sequence. In the lower right, plaques formed by W-SFR or W-H-SFR on monolayers of BSC-1 cells for 48 hours. Below, sequence of the VV TK promoter and the predicted 5′ end of the SFV replicon (arrow).

Insertion in the VV genome was performed in a two-step process (Fig. 3). First, about two-thirds of the SFV genome, encompassing the genes coding for non-structural proteins, together with a dsRed gene cassette was inserted into the TK locus of vaccinia WR. The resulting virus, termed W-RednsTK, was subsequently used for the second step, in which the 3′ end of the replicon cDNA was inserted. The latter portion of the replicon included a GFP gene placed downstream of the SFV sub-genomic promoter.

To test the requirement of SFV sequences in the 3′ end of the replicon, we constructed viruses that incorporated the 70, 170 or 340 nucleotides immediately adjacent to the terminal Poly-A sequence. In addition, a 70 nt-long poly-A sequence was either included or not in each of the constructs. During isolation, virus harboring replicons devoid of the Poly-A sequence failed to produce GFP fluorescence. In contrast, viruses that included Poly-A sequence at the 3′ end of the replicon formed plaques displaying green fluorescence. Notably, GFP fluorescence was similar in cells infected with viruses that included 340, 170 or 70 nucleotides of 3′ terminus sequence. Therefore, the virus which contained 70 nucleotides and the cDNA Poly-A sequence, termed W-SFR, was selected for further study.

### Construction of a Virus Containing the SFV Replicon and the Genes Coding for SFV Structural Proteins

In the original SFV-derived system, the RNA replicon is encapsidated and released from cells expressing SFV structural proteins. This is usually accomplished by transfecting an *in vitro* transcribed helper replicon RNA devoid of the SFV packaging signal. We tested if expression of SFV structural genes from VV recombinant V-Helper would result in packaging of the replicon in W-SFR infected cells. Coinfection of cells with W-SFR and V-Helper resulted in production of SFPs in the medium of infected cells (not shown), indicating that W-SFR-derived replicon RNA was functional for packaging, and further confirmed that replicon packaging could take place in vaccinia-infected cells.

To construct a recombinant virus that would transcribe the replicon and also provide the structural proteins, we assembled the complete replicon in the TK locus as above, but starting from the V-Helper virus containing the SFV structural genes downstream of the F13L gene (Fig. 3). Using the same two-step process described above, virus W-H-SFR was isolated taking advantage of the GFP fluorescence produced by the virus during the isolation process.

### Plaque Formation and SFP Production by W-H-SFR

In the standard VV plaquing assay, virus plaques are usually allowed to develop in cell monolayers maintained under liquid medium. Under those conditions, the size and shape of the virus plaques are good indicators of cell-to-cell virus transmission and extracellular virus release. Some VV strains that are well transmitted locally but release low numbers of infectious virus to the culture medium give rise to round, well defined plaques. In contrast, VV strains which release more extracellular virus typically produce plaques with a comet shape, indicative of secondary infections caused by virus released from the primary plaque. When W-SFR and W-H-SFR were subjected to a plaque assay on BSC-1 cells, a clear difference between the two viruses was noted. W-SFR plaques were of the normal round phenotype, similar to plaques formed by the parental WR virus. In contrast, W-H-SFR produced a comet-shaped plaque phenotype reminiscent of vaccinia viruses producing more extracellular virus like IHD-J (Fig. 3).

**Figure 4:**
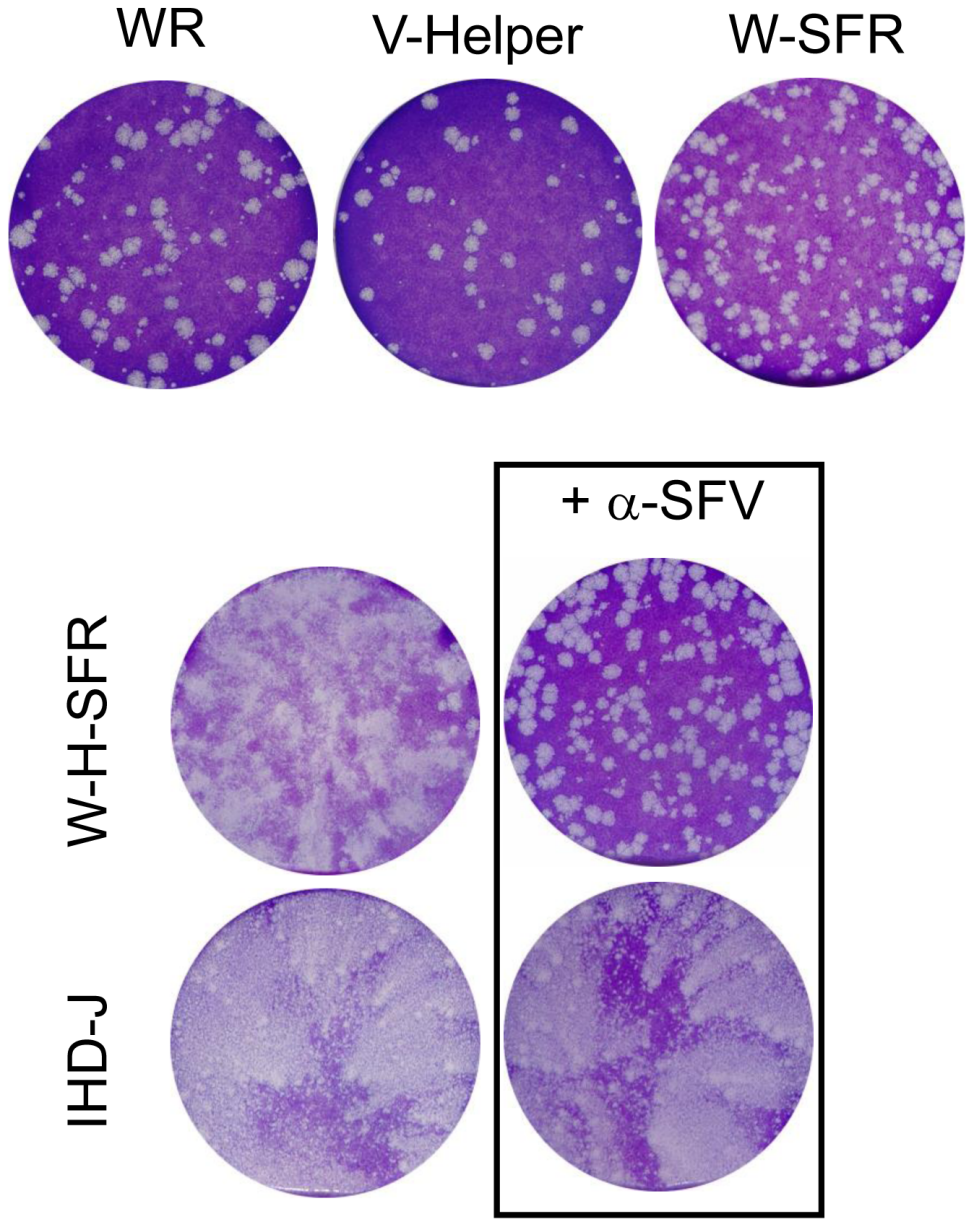
Plaque formation and SFP production by W-H-SFR. Plaque phenotype of the viruses indicated was determined in a standard vaccinia plaque assay on BSC-1 monolayers. Virus plaques were allowed to develop for 48 hours and stained with crystal violet solution. In the cultures treated with anti-SFV antibody, culture medium was replaced at 2 hours post-infection with medium containing polyclonal antiserum to SFV proteins (α-SFV).

We hypothesized that cells infected by W-H-SFR within the primary plaque were releasing SFPs encapsidating the replicon-GFP gene that would infect distant cells within the monolayer. The comet tail would therefore be the result of cythophatic effect caused by those secondary infections. That this was the case was corroborated by the observation that when anti-SFV antibody was included in the culture medium during the plaque assay, comet tails in W-H-SFR plaques were not formed, but instead round plaques developed (Fig. 4).

**Figure 5:**
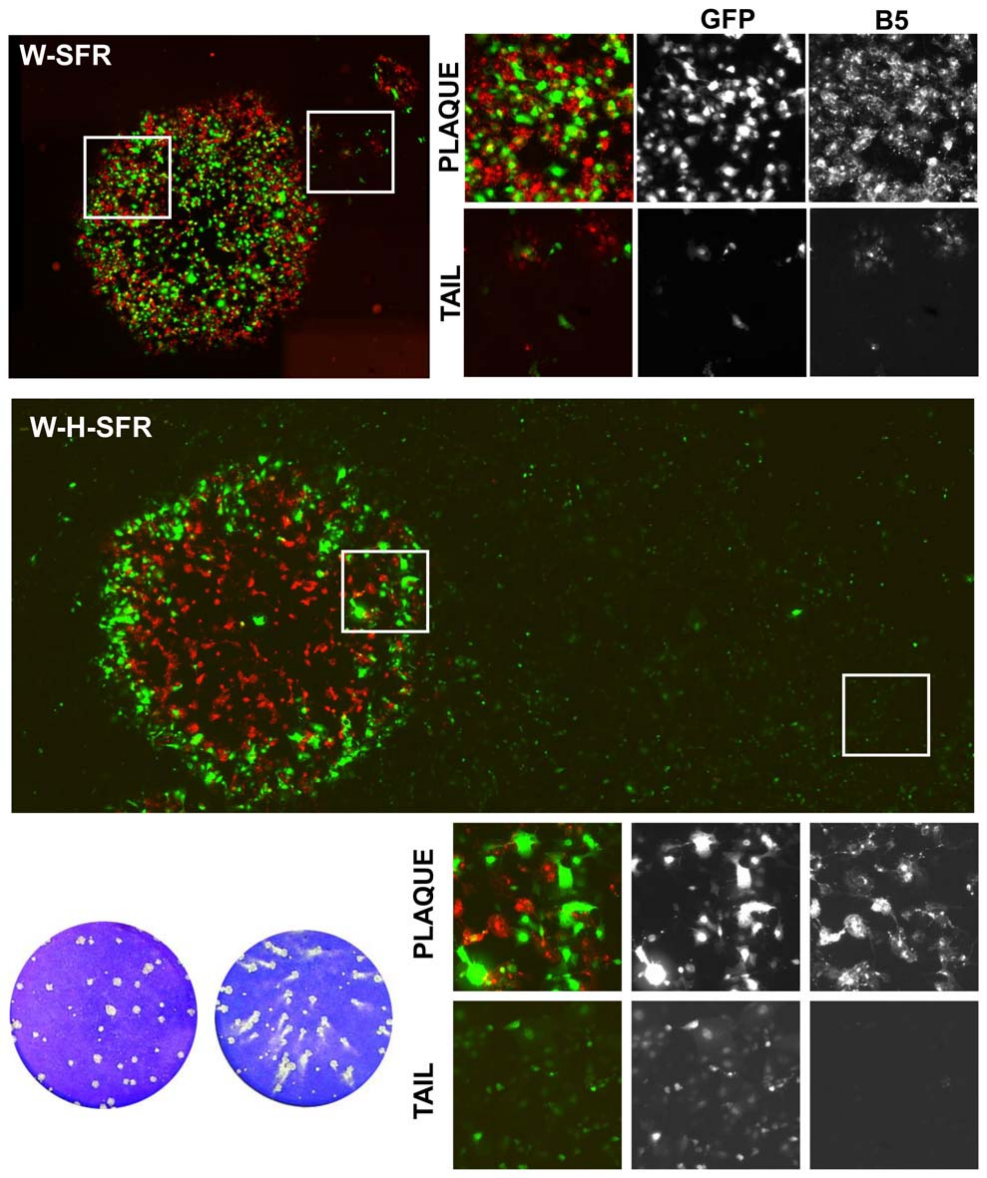
Distribution of cells infected with VV and/or expressing the SFV replicon in BSC-1 cells. BSC-1 cell monolayers were infected with dilutions of W-SFR or W-H-SFR for 48 h, fixed and stained with anti-B5 antibody. Fluorescence within and around virus plaques was visualized in an inverted fluorescence microscope. Merged images result from the combination of monochrome images in red (anti-B5 antibody) and green (direct expression of GFP). Images covering whole plaques and tails were assembled from multiple individual images stitched together as described in the Materials and Methods section. White boxes specify the areas inside (plaque) or outside (tail) the virus plaques that are shown enlarged in the smaller photographs.

In addition to the appearance of comet tails, if infective SFV particles containing the replicon-GFP RNA were being released, a singular distribution of VV and SFP –infected cells within the virus plaque would be expected. To explore this idea, the distribution of cells infected with VV within the virus plaques was visualized by immunofluorescence, and compared with those of cells with replicon-mediated expression of GFP (Fig. 5). In W-SFR plaques, VV staining and the GFP signal appeared within the virus plaque, consistent with the replicon being produced in W-SFR infected cells. Even in the small tails found in some plaques, GFP fluorescence was coincident with VV-positive cells, indicating release of some vaccinia extracellular particles. Significantly, immunofluorescence staining of W-H-SFR plaques revealed a singular distribution (Fig. 5). Within the plaque, the coincidence of VV and GFP positive cells was lower than in the case of W-SFR plaques, being GFP-positive cells more abundant in the outer region of the plaques. Also, while vaccinia staining was present almost exclusively in the primary plaque, GFP-expressing cells were detected both in the area of the primary plaques and in the comet tails. Notably, GFP expressing cells in the comet tail area were not stained by anti-VV antibody, suggesting that they were produced by infecting SFPs (Fig. 5).

**Figure 6:**
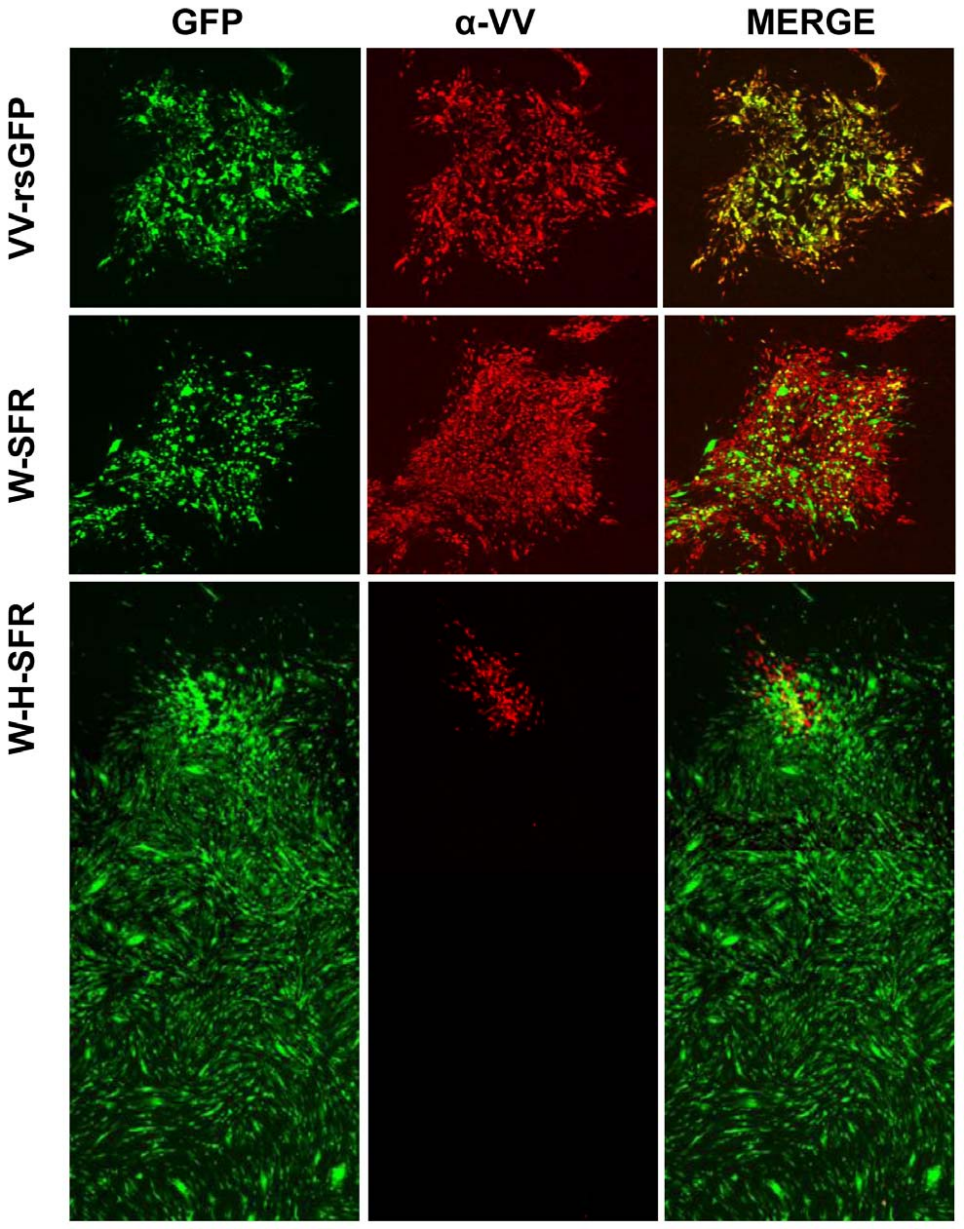
Distribution of cells infected with VV and/or expressing the SFV replicon in BHK-21 cells. BHK-21 monolayers were infected with dilutions of VV-rsGFP, W-SFR or W-H-SFR. At 48 h.p.i, cell monolayers were fixed and stained with polyclonal antiserum to VV proteins. Merged images result from the combination of monochrome images in red (anti-VV polyclonal serum) and green (direct expression of GFP). The larger panel was assembled using overlapping photographs stitched together as described in the Materials and Methods section.

Additionally, a similar plaque assay was carried out in BHK-21 cells, which are highly susceptible for SFV (Fig. 6). Compared with BSC-1 cells, comet tails were much larger in BHK-21 cells, and displayed brighter GFP fluorescence, consistent with the high susceptibility of BHK-21 cells to replicon-mediated expression. Remarkably, the sizes of the primary plaques formed by W-H-SFR were smaller than those formed by W-SFR, probably revealing competition of SFPs with vaccinia virus for cells within the plaque area.

The above results indicate that infection with W-H-SFR, a VV recombinant expressing the SFV replicon and SFV structural proteins, is able to generate infectious SFV particles that, in a second round of infection, promote the expression of a foreign gene.

### Electron Microscopy of W-H-SFR Infected Cells

**Figure 7:**
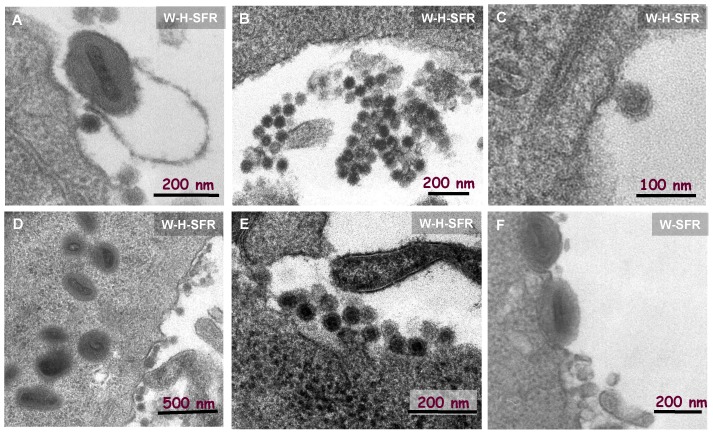
Electron microscopy of W-H-SFR infected cells. Vaccinia virus (particles around 200–250 nm) and smaller, SFV-like particles (SFPs, spherical, approximately 50 nm diameter) were present in W-H-SFR infected cells (Panels A-E). Note that SFPs tend to aggregate in clusters (central panels). A budding image at the plasma membrane can be seen in panel C. An image of the control of W-SFR infected cells (panel F) is shown.

Infectious material in the medium of W-H-SFR was filterable through 100 nm filters that completely removed VV infectivity, suggesting that replicon molecules were encapsidated in small particles like those of SFV. To directly visualize the particles being formed, cells infected by W-SFR or W-H-SFR were analyzed by electron microscopy. In addition of the well known stages of VV morphogenesis, typical alphavirus particles, approximately 60 nm in size, were evident (Fig. 7 A–E). Those Alphavirus-like particles were commonly found in groups or attached to the plasma membrane of cells. Consistently with the morphogenetic pathways for SFV, budding events at the plasma membrane could also be identified. As expected, the appearance of SFV-like particles was dependent on expression of SFV structural proteins, since it was not detected in W-SFR infected cells (Fig. 7-F). In addition, the presence of extracellular SFV particles was not dependent on the exit of VV, since it occurred normally in W-H-SFR infected cells treated with IMCBH, an inhibitor of VV release (not shown).

### Production of SFPs in the Presence of VV Inhibitors

**Figure 8:**
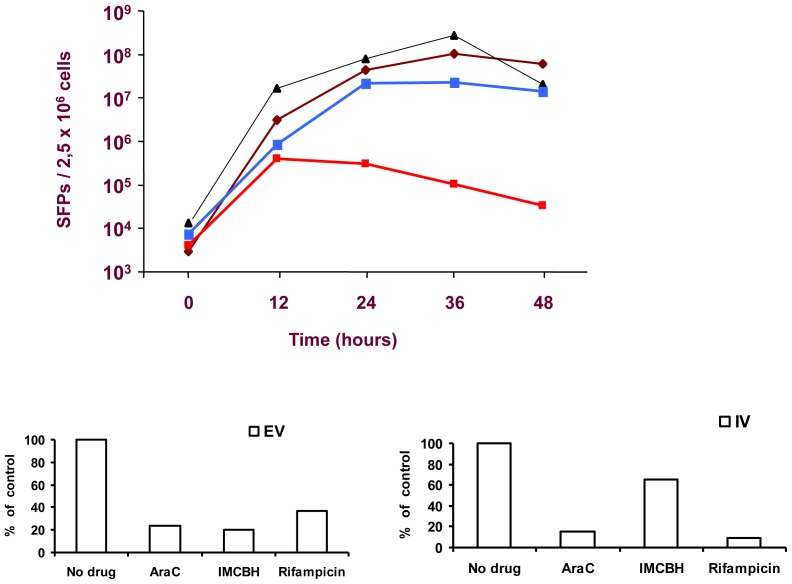
Particle production in the presence of vaccinia virus inhibitors. Hela cells were infected at a m.o.i. of 5-H-SFR and after adsorption, medium was replaced with fresh normal medium (brown rhombs) or medium containing 100 µg/ml AraC (red squares), 10 µg/ml IMCBH (black triangles) or 100 µg/ml Rifampicin (blue squares). Samples of the culture medium were collected every 12 hours, clarified, filtered and stored at 4°C until last point was collected. To determine the titer of SFPs, fresh BHK cells were infected with serial dilutions of the filtered supernatant, and GFP-positive cells were counted 24 hours later in the fluorescence microscope. **Lower panels:** Vaccinia virus Extracellular virus (EV) or cell associated (IV) production. Hela cell monolayers were infected at a multiplicity of 5 pfu per cell with W-H-SFR recombinant virus, and after 1 hour medium was replaced with medium containing AraC (100 µg/ml), rifampicin (100 µg/ml) or IMCBH (10 µg/ml). Virus was harvested at 24 hpi and Vaccinia virus titers in the culture medium (EV) (left panel) and cell lysates (IV) (right panel) were obtained by plaquing in BSC-1 cell monolayers and are represented relative to the control with no inhibitor.

We studied the effect of VV inhibitors in the production of SFPs from W-H-SFR infected cells (Fig. 8). HeLa cells infected with W-H-SFR produced increasing amounts of SFPs up to 36 hours post-infection. As expected, SFP production was only slightly affected by Rifampicin or IMCBH, two specific inhibitors of VV morphogenesis and release that do not inhibit viral gene expression. In contrast, a marked decrease was noted by treatment with AraC, an inhibitor of DNA replication that blocks VV intermediate and late gene expression. Since AraC should not affect RNA replication, we consider it likely that the effect of AraC is the consequence of the lower expression of SFV structural proteins, which is dependent, in W-H-SFR, on a VV early/late promoter. As the inhibition of VV DNA replication abolishes late gene expression, it is expected that SFV structural proteins will be limiting at late times, in conditions where replicon amplification can continue unabated.

### Absence of Viable SFV

One important consideration in the design of alphavirus vectors is the possibility of generating viable viruses by RNA recombination, a prospect imposing important biosafety limitations to the system. In the established replicon/helper system, replication-proficient viruses were detectable in the stocks in ratios to SFPs of 10^−3^ to 10^−6^ [12]. This frequency has been lowered by using several strategies, including the mutation of the spike glycoprotein, the use of two different Helper RNAs, promoterless helpers or the inclusion of miRNA targets in the helper RNA [12], [13], [14], [15].

The system described here should hamper the production of replicating SFV, since the structural proteins are produced via a VV RNA with no homology to the replicon and lacking 5′ and 3′ non-translated sequences of the subgenomic RNA. We tested if replication competent SFV were present in SFP stocks generated from W-H-SFR. To this aim, 10^7^ SFPs were used to infect BHK-21 cell monolayers, and after 48 hours, the supernatant was harvested and used to infect fresh BHK-21 cell monolayers, in an attempt to detect any replicating SFV. Consistently, in three independent experiments, we failed to detect replication-competent SFVs in our SFP stocks, suggesting a significant improvement over the replicon/helper RNA system.

## Discussion

The strategy outlined in this work demonstrates that a single vaccinia virus can serve as a vehicle to produce Alphavirus particles in infected cells. Due to the technical complexity and considerable difficulties to produce Alphavirus replicons in a cost-effective manner, it may be desirable to generate and package a self-amplifying replicon *in vivo*. This strategy not only obviates recombinant SFP production problems, but also provides a potential means to greatly enhance the efficacy of Alphavirus or Poxvirus -based vaccines.

The concept of combining two viral vectors may be used to exploit the benefits of each of the individual vectors involved. So far, a number of different combinations have been described, including Adeno-Retrovirus [16], [17], [18], [19], [20] and SFV – Retrovirus [21], [22], [23]. Many of these systems require the coinfection of several trans-complementing virus recombinants or trans-complementing cell lines, since the virus recombinants used do not accommodate all the genetic information required. Poxviruses constitute an attractive alternative to those since, in addition to their well-known capabilities as vaccines and reliable expression systems, they allow the insertion of large and multiple DNA sequences. Taking advantage of these features, VV-retrovirus hybrid vectors have been developed previously, and shown to be capable of producing transduction-competent retroviral particles [24], [25] [26], [27], [28].

Vaccinia virus offers several advantages to act as a “shuttle” vector for an RNA virus vector. Most important, the coding capacity of the vector and the well characterized transcriptional control of vaccinia genes make it possible to express both the replicon and the genes coding for the structural proteins from different locations in the genome. In the past, a vaccinia virus recombinant expressing a Venezuelan Equine Encephalitis replicon from a T7 promoter, in which the replicon RNA could be transcribed after coinfection with a second recombinant expressing the T7 RNA polymerase, has been described [29]. There are two main differences in our system with respect to the previous one. First, here the transcription of both the replicon and the genes coding for the packaging protein are dependent on VV promoters, and therefore expression of the T7 RNA polymerase is not required. Second, the packaging proteins are expressed from the same VV recombinant, and therefore no coinfections are required to trigger the replicon and the production of suicide SFPs. Those facts are crucial for the use of this, or similar viruses, as immunizing agents *in vivo*, with the aim of using VV as a shuttle to generate suicide SFPs inside the injected tissues.

One potential improvement of the system described here over existing SFV expression systems is the low generation of replication competent SFV. To prevent recombination leading to replicative SFV, we designed the constructs to avoid the occurrence of any repeated sequences between the replicon and the gene coding for the SFV structural proteins. We anticipated that generating viable SFV would be an extremely rare event, since the VV mRNA coding for SFV structural genes in our recombinant lacks a SFV subgenomic promoter, as well as any non-coding sequences at the 3′ end. Thus, reconstruction of a viable SFV genome would need two precise recombination events in regions with no homology. There may be additional reasons for the absence of SFV in our preparations. For instance, if the mRNA for the SFV structural proteins behaves like a normal VV transcript and does not interact with the SFV replicase, physical interaction with the replicon RNA may not be favored, and they might be compartmentalized in different subcellular locations as a consequence of the different transcription/replication strategy for the two viral systems.

Another feature related to the safety of the vector is that the system could be applied to replication-defective Poxvirus vectors (like MVA) that do not expand within the tissues in mammalian hosts. In that situation, even with limited spread of the poxvirus, the combined vector might maintain a higher level of expression due to the second cycle of expression in the SFP-infected cells. This “expanded expression” effect is likely to be dependent on the susceptibility of cells to the SFV, as exemplified by plaque assays in different cell lines (compare the GFP expression in Figs. 5 and 6).

One interesting question is how this double expression might affect the immunological response to the relevant antigen. Up to date, a number of approaches have been used to enhance the response to the foreign antigen in a context of multiple vector-specific antigens. For instance non-replicating or growth-restricted vaccines such as plasmid DNA or VV MVA are often used in combination with other heterologous vectors delivering a common antigen in prime-boost regimens. It is tempting to speculate that the two vector system described here, that directs expression of the antigen in two different cellular contexts (the VV-infected cell and the SFP-infected cell) might have consequences with respect to the immune responses to the antigen. Further, this system could be used in combination with additional immunizing agents to further increase the responses.

## Materials and Methods

### Cells and Viruses

BSC-1 cells were grown in Eagle’s minimal essential medium (EMEM) supplemented with 0.1 µg/ml penicillin, 0.1 µg/ml streptomycin, 2 mM L-glutamine (Bio Whittaker) and 5% fetal bovine serum (FBS). BHK-21 cells (ATCCCCL10) were grown in BHK-21 Glasgow minimal essential medium (Glasgow-MEM, GibcoBRL) containing 5% FBS, 3 g/ml tryptose phosphate broth, 0.01 M HEPES and supplemented with antibiotics and glutamine. Virus infections were carried out in medium containing 2% FBS. Plaque assays and crystal violet staining was carried out as described [30] [31].

Vaccinia virus expressing β-Glucuronidase (V-Gus) was isolated by insertion of the β-Glucuronidase gene downstream of the F13L gene under the control of a synthetic early/late vaccinia promoter. For this, plasmid pRB21-βGus [32] was transfected into cells infected with virus vRB12 [33], and recombinant viruses were subsequently isolated by plaque selection [34]. Vaccinia virus expressing Sp6 DNA polymerase vSIMBE/L [35] was kindly made available by B. Moss.

### Generation of Semliki Forest Virus Suicide Particles Packaging Replicons

Semliki Forest Particles (SFPs) were generated in cell cultures by electroporation of plasmids containing the SFV replicon downstream of the SP6 promoter together with pSFV-Helper1 helper plasmid [6]. *In vitro* RNA synthesis, transfections and SFV infections were performed as described [11], [36].

### Construction of Plasmid pSFV-GFP

The GFPst gene was obtained by PCR from the virus W-GFPs_65_T [37] [38] with oligonucleotides pRB21–5′BamHI 5′-TTTTTTTTTGGATCCTAAATAAGGAAT-3′ and RB21.3 Smal 5′-AAAATTATTTACCCGGGCCTCCATGG-3′, and cloned into the plasmid pSFV1 (a generous gift of Henrik Garoff, Karolinska Institutet) between sites BamHI/SmaI. SFPs for expression of LacZ or GFP were obtained by transfection of in vitro transcribed RNA from plasmids pSFV3-lacZ ([6] a generous gift of Henrik Garoff) or pSFV-GFP.

### Construction of V-Helper

Plasmid pRB-Helper was constructed by inserting the genes for SFV structural proteins (C-p62-6K-E1) downstream of a synthetic VV early/late promoter in expression plasmid pRB21 [34]. SFV sequences were obtained by digestion of pSFV-Helper1 and cloned in pRB21 in two steps. First, an EcoRI-HindIII fragment (pSFV-Helper1 coordinates 2653–5281) was ligated into the EcoRI/HindIII sites in pRB-21. Subsequently, an EcoRI fragment (pSFV-Helper1 coordinates 1298–2653) was cloned into the resulting single EcoRI site.

Vaccinia virus recombinant VV-Helper was isolated by a plaque size selection method [34]. Plasmid pRB-Helper was transfected into cells infected with v-RB12 virus, and a virus with large plaque phenotype was isolated through successive rounds of plaque purification.

### Generation of Recombinant Viruses W-SFR and W-H-SFR

Recombinant viruses with the SFV replicon inserted in the VV thymidine kinase (TK) locus were isolated in two steps. First, intermediate viruses were isolated by recombination of the viral genome with plasmid pRednsTK, which contains two-thirds of the SFV replicon cDNA and a dsRed2 cassette flanked by recombination flanks for insertion into the TK locus (see Fig. 3). In a second step, the foreign gene was inserted in place of the dsRed2 cassette.

Plasmid pRednsTK was isolated as follows. Recombination flanks for the TK locus were amplified from vaccinia WR DNA. SFV sequence was assembled precisely at the transcriptional start nucleotide of the VV TK promoter, by recombinant PCR using two fragments amplified with oligonucleotides.

TK-LL, 5′-GCTTTTGCATGCAATAAATGGATCACAA-3′ and P-TK-1R 5′-GAATTAATTATTGTTCACTTTATTCGACT-3 (VV left TK flank, amplified on WR virus DNA) and p TK 1F 5′-GAATAAAGTGAACAATAATTAATTCTCGAC-3′ and SFV 700 XhoI 5′-CACCTGCTCGAGGGCCCAGTTTGTG-3′. This PCR fragment spans the 5′ end of the SFV cDNA, which included the BsiWI site at coordinate 514 of pSFV-1 used later to clone additional SFV sequences.

Right VV TK flank was amplified with oligonucleotides TK_RL_ 5-TTGGGTGAGGATCCCGAGATAGAAATAA-3′ and TK_RR_ 5′-TTTTTGATGCATCGTAGATATTCCTCATC-3′. An intermediate plasmid containing the TK flanks and the 5′ end of the SFV genome was termed pRedTK.

SFV sequences were obtained as follows: First, a TTTTTGT sequence, (which constitutes a VV early transcription termination signal) present in the ns4 gene of the SFV replicon was mutated to TTTCTGT in plasmid pSFV-1. Subsequently, a 6887 bp fragment containing the most of the sequence of the non-structural genes of the SFV replicon was obtained from this plasmid by digestion with BsiWI and BamHI (coordinates 514 to 7404 in pSFV-1) and was inserted into the corresponding sites in plasmid pRedTK, generating plasmid pnsTK. Then, a fragment containing dsRed gene under a synthetic early/late promoter was digested with BamHI and BglII from pRBdsRed2 [39] and inserted in BamHI site of plasmid pnsTK. The resulting plasmid, pRednsTK, contains a dsRed cassette and most of the SFV replicon flanked by WR TK flanking sequences.

Plasmid pMix-f70An, designed for insertion of foreign genes in the sub-genomic region of the SFV replicon, was derived from pRednsTK by substituting a SphI-HindIII fragment containing the nsP1–4 genes, the dsRed2 cassette and the TK left flank by a fragment encompassing a portion of nsP4 gene and the GFP marker gene placed downstream of the SFV subgenomic promoter. This fragment was amplified by PCR from pSFV-rsGFP using oligonucleotides ns-4 f 5′-CGGTCGGCATGCAACGAGATGTCA-3′ and SFV GFP Hind R 5′-GGGATGTAATAAGCTTAATTACCCGG-3′. Then, a fragment spanning 140 nt of the replicon 3′ sequence (including a 70 nucleotide-long polyA tail) was amplified by PCR from pSFV-1 using oligonucleotides F70Xma 5′-GGCAATACCCGGGAGCTTACATAAG-3′ and FStuRPoliA 5′-GCGCGTAGGCCTATTCATTAATGCA-3′ and inserted in the Xma-I and Stu-I sites in pMix using the corresponding sites included in the oligonucleotides. The resultant plasmid, pMix-f70An contains a portion of the nsP4 gene as the left recombination flank, the GFP gene under the SFV subgenomic promoter, 70 nucleotides of 3′ sequence, plus a 70 nt polyA sequence of the SFV replicon and the right flank of TK gene.

Plasmid pRednsTK was used to generate VV recombinants containing the ns1–4 genomic region of the SFV replicon. Thus, pRednsTK was transfected into cells infected with WR virus or VV-Helper to generate recombinant viruses W- RednsTK or W-H-RednsTK (Figure 3). These recombinant viruses were subsequently used for insertion of the 3′ end of the replicon.

The final combined vectors W-SFR and W-H-SFR were isolated by recombination of pMix-f into the W-RednsTK or W-H-RednsTK recombinant viruses, respectively (See scheme in Fig. 3). Recombinant viruses were isolated by plaque isolation, by identifying plaques under the fluorescence microscope by GFP expression or absence of dsRed2 expression.

### Quantitation of β–Galactosidase and β–Glucuronidase

BHK-21 cells grown in 12-well plates were mock infected, infected with WR or with vaccinia virus recombinant vSIMBE/L which express Sp6 RNA polymerase at a moi of 5 PFU/cell. One hour later, the cells were transfected with 2 µg of pSFV-LacZ plasmid using Lipofectin, following manufacturer’s protocols. At 5 h posttransfection medium was replaced with fresh medium. At 36 h the cells were lysed in 300 µl of lysis buffer (1% NP40, 50 mM Tris-HCl pH 7.6, 150 mM NaCl, 2 mM EDTA, 1 µg/ml PMSF), for 10 min at 4°C. β–Galactosidase or β–Glucuronidase in the cell extracts was measured using ONPG (Sigma N1127) or 4-NPG (Sigma N1627) as substrates, respectively. After a 20 min incubation at 37 °C the enzymatic reaction was followed by color development at 414 nm. 100 µl of diluted lisate was tested addition 100 µl of BufferZ-ONPG2X [11].

### Electron Microscopy

Monolayers of Hela cells in p100 culture plates were infected with W-H-SFR virus at a moi of 5 pfu/cell. At 24 hpi, the culture medium was removed and the cells were fixed by adding a solution of 2% glutaraldehyde, 1% tannic acid, and 0.4 M HEPES (pH 7.2) directly to the monolayer. After 2 hours of fixation at room temperature, cells were scraped and pelleted by centrifugation at 2000 rpm for 5 min. Cells were resuspended in 1 ml of HEPES buffer and were included in Epon 812 resin as described [40]. Ultrathin sections were obtained with a Ultracut microtome, which were deposited on copper grids coated with a film of coloidon/carbon. Finally, the preparations were contrasted with lead citrate 0.2% under nitrogen and examined by electron microscopy.

### Fluorescence Microscopy

To generate stocks of single-cycle Semliki Forest virus particles (SFPs), BHK 21 cells were infected with W-H-SFV at a moi of 5 pfu/cell. At 24 h.p.i, media were collected, clarified (centrifuged 5 minutes at 2000 rpm) and filtered through a 0.1 µm filter to remove vaccinia virus. Filtered media were aliquoted and kept as SFP stocks. In order to measure the titer of SFPs, cell culture supernatants were titrated on fresh monolayers of BHK-21 or BSC-1 cells grown to 70–80% confluence. Different concentrations of the filtered medium were used to infect cells seeded in 6-well plates. After a 2 hour adsorption period, the cells were incubated for at least 24 hours. Finally, the titer of SFV particles was estimated by counting cells displaying GFP fluorescence under a Nikon Eclipse TE2000-E inverted fluorescence microscope.

To visualize large virus plaques in single images, a number of overlapping microscopy images were acquired from individual virus plaques. These images were stitched together using a Grid/Collection Stitching plug-in for ImageJ [41].
